# Polyfunctional donor-reactive T cells are associated with acute T-cell-mediated rejection of the kidney transplant

**DOI:** 10.1093/cei/uxad041

**Published:** 2023-04-18

**Authors:** Nicolle H R Litjens, Amy C J van der List, Mariska Klepper, Fréderique Prevoo, Karin Boer, Dennis A Hesselink, Michiel G H Betjes

**Affiliations:** Erasmus MC Transplant Institute, Department of Internal Medicine, Division of Nephrology and Transplantation, University Medical Center Rotterdam, Rotterdam, The Netherlands; Erasmus MC Transplant Institute, Department of Internal Medicine, Division of Nephrology and Transplantation, University Medical Center Rotterdam, Rotterdam, The Netherlands; Erasmus MC Transplant Institute, Department of Internal Medicine, Division of Nephrology and Transplantation, University Medical Center Rotterdam, Rotterdam, The Netherlands; Erasmus MC Transplant Institute, Department of Internal Medicine, Division of Nephrology and Transplantation, University Medical Center Rotterdam, Rotterdam, The Netherlands; Erasmus MC Transplant Institute, Department of Internal Medicine, Division of Nephrology and Transplantation, University Medical Center Rotterdam, Rotterdam, The Netherlands; Erasmus MC Transplant Institute, Department of Internal Medicine, Division of Nephrology and Transplantation, University Medical Center Rotterdam, Rotterdam, The Netherlands; Erasmus MC Transplant Institute, Department of Internal Medicine, Division of Nephrology and Transplantation, University Medical Center Rotterdam, Rotterdam, The Netherlands

**Keywords:** donor-reactive T cells, CD137-expressing T cells, acute T-cell mediated rejection, polyfunctionality

## Abstract

Acute T-cell-mediated rejection (aTCMR) still remains a clinical problem after kidney transplantation despite significant improvements in immunosuppressive regimens. Polyfunctional T cells, i.e. T cells producing multiple pro-inflammatory cytokines, are believed to be the most relevant T cells in an immune response. The aim of this study was to determine whether polyfunctional donor-reactive T cells are associated with aTCMR. In a case–control study, 49 kidney transplant recipients with a biopsy-proven aTCMR in the first year after transplantation were included, as well as 51 controls without aTCMR. Circulating donor-reactive T cells were identified by the expression of CD137 after short-term co-culture with donor antigen-presenting cells. Polyfunctional donor-reactive T cells were further characterized by dissection into different T-cell subsets encompassing the spectrum of naïve to terminally differentiated effector T cells. Prior to kidney transplantation, proportions of donor-reactive CD4+ (0.03% versus 0.02%; *P* < 0.01) and CD8+ (0.18% versus 0.10%; *P* < 0.01) CD137++ T cells were significantly higher in recipients with a biopsy-proven aTCMR versus non-rejectors. Polyfunctionality was higher (*P* = 0.03) in this subset of CD137-expressing T cells. These cells were predominantly of the EM/EMRA-phenotype, with polyfunctional donor-reactive CD137++CD4+ T cells predominantly co-expressing CD28 whereas approximately half of the polyfunctional CD137++CD8+ T cells co-expressed CD28. In addition, at the time of aTCMR, polyfunctional donor-reactive CD137++ CD4+, but not CD8+, T cells, were specifically decreased by 75% compared to before transplantation in recipients with as well as those without an aTCMR. Prior to transplantation, the proportion of polyfunctional donor-reactive CD137++ T cells is associated with the occurrence of a biopsy-proven aTCMR within the first year after transplantation.

## Teaser

Acute TCMR remains a clinical problem after kidney transplantation despite significant improvements in immunosuppressive regimens. Poly-functional (producing ≥2 cytokines) T cells are believed to be the most relevant T cells in an immune response. Prior to kidney transplantation proportions of poly-functional CD137 high expressing donor-reactive CD4+ and CD8+ T cells are higher in recipients experiencing an acute TCMR within the first year after transplantation compared to those who remained free of rejection.

## Graphical Abstract

**Graphical Abstract:**
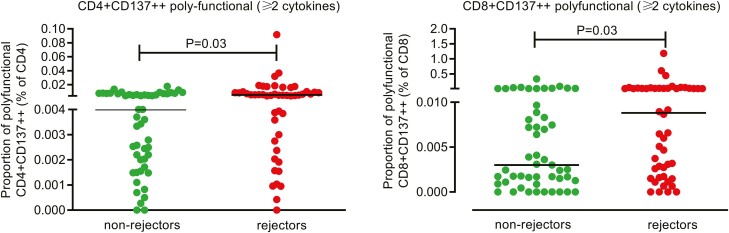

## Introduction

Despite significant improvements in immunosuppressive drug regimens, acute T-cell mediated rejection (aTCMR) still poses a significant threat to allograft survival in 5–15% of the kidney transplant recipients in the first year [1]. There is a need to develop robust assays allowing for quantification of donor-reactive T cells prior to transplantation which assist in assessing a kidney transplant recipient’s risk for the development of aTCMR. Assays to characterize direct donor-reactive T cells have evolved from bulk assays to single-cell assays. Currently, the enzyme-linked immunospot (ELISPOT) assay, in particular, the interferon-gamma (IFN-γ) ELISPOT assay is considered the most powerful tool to assess proportions of donor-reactive T cells at various time points in the setting of kidney transplantation. Pre-transplant assessment of frequencies of IFN-γ producing cells has been shown to be associated with the risk of aTCMR [2]. However, sensitivity and specificity differ greatly among various studies, and validation in other independent study cohorts is poor [3–5].

We have developed a multi-parameter flow cytometry-based assay utilizing expression of the activation-induced marker (AIM) CD137 following short-term stimulation with donor cells [6]. This assay allows for analysis of donor-reactive T cells recognizing donor antigens via the direct pathway of allopresentation. Moreover, detailed phenotypic and functional characterization of both CD4+ and CD8+ T cells at the single-cell level is possible [6–8]. Simultaneous assessment of different cytokines allows for identification of polyfunctional T cells, for example, T cells able to produce multiple pro-inflammatory cytokines (e.g. interleukin (IL)-2, IFN-γ and tumor-necrosis factor-alpha (TNF-α)). Recently, we used this multi-parameter flow-cytometry-based CD137 assay to analyze donor-reactive T cells in a cohort of stable kidney transplant recipients prior to and 3–5-years after kidney transplantation. A significant decrease in polyfunctional donor-reactive CD137-expressing CD4+ T cells was found to be associated with donor-specific hypo-responsiveness, a phenomenon occurring late after transplantation [7, 9]. Polyfunctional T cells are believed to be the most relevant T cells in an immune response and for instance offer correlates for vaccine efficacy or immune-mediated disease progression [10]. Therefore, we tested the hypothesis that prior to kidney transplantation, the frequency of circulating polyfunctional donor-reactive T cells is associated with the risk of aTCMR and that these cells may serve as a biomarker for TCMR risk stratification.

## Material and methods

### Study population

In this case–control study, 49 kidney transplant recipients with a biopsy-proven aTCMR, occurring within the first year after kidney transplantation (early rejection), were included as cases. The control group consisted of 51 randomly selected kidney transplant recipients without a rejection and no need for a biopsy, matched for the period of transplantation (i.e. 2011–2021). Study population characteristics are depicted in Table 1. Peripheral blood mononuclear cells (PBMCs) were collected prior to transplantation. For six of the 49 kidney transplant recipients, PBMCs were also sampled at the time of aTCMR (8 (2–341) days after transplantation). Likewise, we collected PBMCs from four non-rejectors at the same time interval as for those that experienced a rejection (90 (7–365) days after transplantation). Biopsies were scored according to the Banff 2015 criteria [11]. aTCMR was subdivided into acute tubulo-interstitial rejection (aTCMR I) or acute vascular rejection (aTCMR II). The study was approved by the Medical Ethical Committee of the Erasmus MC and all participating kidney transplant recipients gave written informed consent to participate in this study (MEC-2012-022 or MEC-2018-035). This study was conducted in accordance with the Declaration of Helsinki and the Declaration of Istanbul and in compliance with International Conference on Harmonization/Good Clinical Practice regulations.

**Table 1.** Study population characteristics.

Kidney transplant recipients	No rejection (*n* = 51)	Rejection (*n* = 49)	*P*
Age recipient (yrs)	55 (19–79)	63 (28–77)	<0.01
Age donor (yrs)	55 (26–77)	63 (35–76)	<0.01
Male gender recipient, % (*n*)	60.8 (31)	59.2 (29)	0.89
Male gender donor, % (*n*)	49 (25)	44.9 (22)	0.67
CMV-seropositivity recipient, % (*n*)	51 (26)	59.2 (29)	0.32
CMV-seropositivity donor, % (*n*)	51 (26)	57.1 (28)	0.48
CMV-serostatus donor/recipient			0.22
−/−	27.4 (14)	16.3 (8)	
+/−	21.6 (11)	20.5 (10)	
−/+	21.6 (11)	26.5 (13)	
+/+	29.4 (15)	36.7 (18)	
Mismatch HLA class I	2 (1-4)	3 (0–4)	0.76
Mismatch HLA class II	1 (0–2)	1 (1–2)	0.51
Mismatch HLA class I and II	4 (2–6)	5 (1–6)	0.97
PRA current (%)	0 (0–79)	0 (0–97)	0.65
PRA historic (%)	4 (0–100)	4 (0–97)	0.79
ABO incompatible, % (*n*)	0 (0)	4.1 (2)	0.04
Re-transplantation, % (*n*)	5.9 (3)	8.2 (4)	0.58
Underlying kidney disease			0.50
Nephrosclerosis/atherosclerosis/hypertension	21.6 (11)	24.5 (12)	
Primary glomerulopathies	23.5 (12)	30.6 (15)	
Diabetes	19.6 (8)	18.4 (9)	
Urinary tract infections/stones	0 (0)	2 (1)	
Reflux nephropathy	7.8 (4)	0 (0)	
Polycystic kidney disease	9.8 (5)	8.2 (4)	
Other	17.6 (9)	14.3 (7)	
Unknown	3.9 (2)	2 (1)	
Pre-emptive kidney transplantation, % (n)	35.3 (18)	34.7 (17)	1.00
Type of transplantation			<0.01
Living-unrelated	37.3 (19)	46.9 (23)	
Living-related	41.1 (21)	14.3 (7)	
Deceased	21.6 (11)	38.8 (19)	
Time to biopsy-proven rejection (days post-transplant)		8 (2–341)	
TCMR I		26 (5–341)	
TCMR II		8 (2–259)	
Acute T-cell-mediated rejection type*, % (*n*)			
TCMR I		35 (17)	
TCMR II		65 (32)	

^*^Biopsy-proven T-cell mediated rejection within first year after transplantation.

Values are median (min–max) or proportion and number, respectively.

### PBMCs isolation

Ficoll-Paque Plus (GE Healthcare, Uppsala, Sweden) was used to isolate PBMCs from lithium-heparinized blood and stored at −150°C until further use at 10 million PBMCs/vial as described previously [12].

### CD3+ T-cell depletion stimuli

CD3+-depleted donor and third party (with an equal but different number of HLA-mismatches with the tested recipient as the donor) PBMCs or spleen cells were used to stimulate recipient PBMCs. Cells were thawed and depleted for CD3+ T cells following labeling with CD3 microbeads according to manufacturer’s instruction (Miltenyi Biotec, Bergisch Gladbach, Germany). The efficiency of CD3+ T-cell depletion was evaluated using flow cytometry (>95%). CD3-depleted stimuli were allowed to rest for at least 6 h at 37°C before use [6].

### Functional and phenotypic characteristics of donor-reactive T cells

The multi-parameter flow cytometry-based CD137 assay was used, as described previously [6] Briefly, following thawing, viability assessment using trypan blue, and 6 h of rest at 37°C, recipient PBMCs were stimulated with CD3-depleted donor or third-party cells. Stimulation was done at a 1:0.5 ratio for 15 h in the presence of anti-CD49d (1 μg/ml, Becton Dickinson, Erembodegem, Belgium) to allow for the detection of donor-reactive T cells with a high activation threshold and a protein secretion inhibitor (eBioscience, ThermoFisher Scientific, Bleijswijk, the Netherlands).

**Figure 1.**
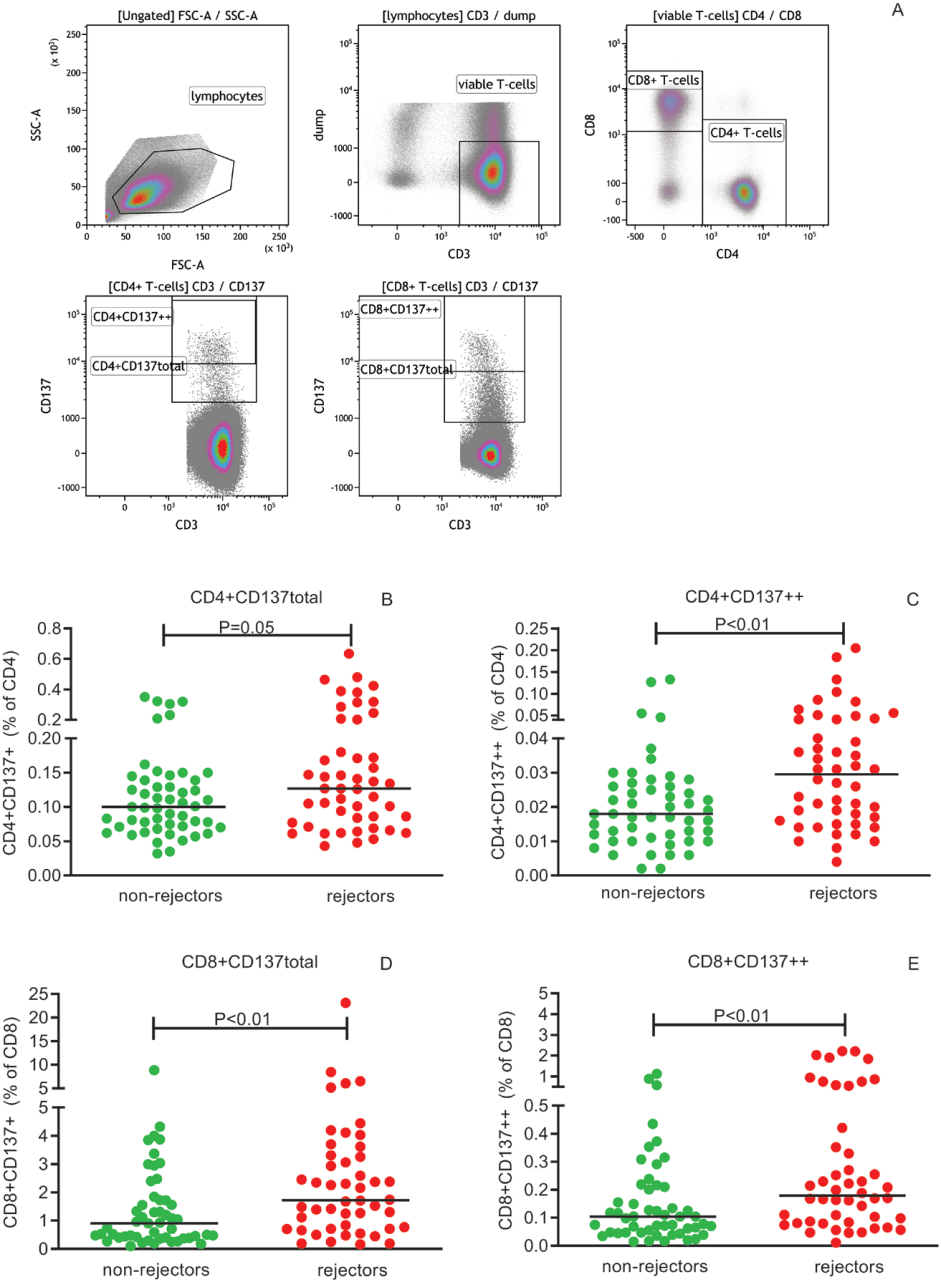
Identification of donor-reactive (CD137-expressing) T cells. A typical flow cytometric example of the identification of donor-reactive T cells is given in **A**. Briefly, lymphocytes are selected based on their forward/sideward characteristics in the first plot, these lymphocytes are then plotted to identify viable (FVS780−) CD3+ T cells, thereby excluding monocytes (CD14+), B cells (CD19+) and NK cells (CD56+) using a DUMP channel. CD4+ and CD8+ T cells were identified within the viable CD3+ T cells and subsequently plotted to identify CD137 (“total”) and CD137 high (“++”)-expressing T cells. In **B–E**, proportions of CD137− as well as CD137 high-expressing CD4+ (B and C, respectively) and CD8+ (D and E, respectively) T cells prior to transplantation are depicted for recipients experiencing a TCMR within the first year after kidney transplantation (rejectors) and those without (non-rejectors). The horizontal line depicts the median value. *P* < 0.05 is statistically significant

Stimulation was stopped by adding a final concentration of 2 mM ethylenediaminetetraacetic acid (EDTA) and incubation for 15 min at room temperature. After washing, cell surface and intracellular staining were performed as described elsewhere [6]. The panel of monoclonal antibodies used for characterization of CD137-expressing T cells is given in Supplementary Table S1. A typical flow cytometric example of the identification of donor-reactive T cells is given in Fig. 1A.

To exclude unwanted cells from our analysis, a DUMP channel was included for cells stained with the live/dead discrimination marker fixable viability stain (FVS)-780 and cells positive for CD14, CD19, and CD56. Moreover, antibodies directed to CD3, CD4, and CD8 were used to allow for identification of the different T cells. The antibody directed to CD137 allowed for identification of antigen-reactive, i.e. donor-reactive T cells [6].

Furthermore, the different T-cell subsets were identified using an antibody directed against CD45RA in combination with CCR7. Naïve T cells were identified as CCR7+CD45RA+, central memory (CM) as CCR7+CD45RA−, effector memory (EM) as CCR7−CD45RA− and terminally differentiated effector memory (EMRA) as CCR7-CD45RA+. The antibody directed against CD28 allowed more broad discrimination between less differentiated cells (CD28+) and more differentiated cells (CD28null) [6]. Proportions of cytokine-producing cells were determined using the following cytokines: interleukin (IL)-2, interferon (IFN)-γ, and tumor necrosis factor (TNF)-α. Polyfunctional T cells were identified as T cells expressing two or more of these pro-inflammatory cytokines. In addition, fractions of degranulated cells were evaluated by assessing CD107a (lysosomal-associated membrane protein-1; LAMP-1) positivity. CD107a is a marker associated with cytotoxic potential [13].

Samples were measured on the BD FACS Symphony™ A3 light flow cytometer (BD; 4 laser configuration, i.e. Violet (8 detectors)-Blue (6 detectors)-Yellow Green (4 detectors)-Red (3 detectors)). We acquired 0.5-1 million viable T cells and aimed to store more than 10 events of CD137 high-expressing CD4+ or CD8+ T cells per measurement. Samples with less than 10 events were excluded from further analysis. Analysis was done using Kaluza software version 2.1 (Beckman Coulter BV, Woerden, the Netherlands).

### Statistical analysis

Statistical analyses and graphs were performed and created using GraphPad Prism (version 9.0.0). Normally distributed data are expressed as mean (95%CI); non-normally distributed data as median and interquartile range (IQR) or min–max. Continuous variables of the study population were compared using the unpaired *t*-test or Mann-Whitney *U*-test, as appropriate. Discrete data were analyzed as frequencies with Chi-square test or Fisher’s exact test. Demographic and patient characteristics are depicted as median and IQR, number and proportion of total, respectively. A multivariate analysis was performed in SPSS® version 28.0.1.0 for Windows (SPSS Inc., IL, USA) to evaluate whether significant clinical and/or demographic characteristics for the study population could account for the variation observed in proportions of donor-reactive triple, double, or single cytokine producing CD137++ CD4+ and CD8+ T cells.

## Results

### Study population characteristics

The study population characteristics are depicted in Table 1. Rejectors were older than non-rejectors, i.e. median (min–max) age amounted to 63 (28–77) years versus 55 (19–79) years (Table 1). The donors were older in the rejector group compared to those in the non-rejector group, 63 (35–76) years versus 55 (26–77). Rejectors more often received a kidney from a deceased donor (Table 1). Seventy-five percent of rejectors developed an aTCMR within first 3 months after kidney transplantation. Sixty-five percent of the rejectors developed a vascular type of acute T-cell-mediated rejection (aTCMR II) whereas 35% developed a tubular-interstitial acute T-cell-mediated rejection (aTCMR I) (Table 1). All patients received induction therapy with basiliximab followed by the standard triple immunosuppressive regimen at our center consisting of tacrolimus, mycophenolate mofetil, and glucocorticoids.

### Rejectors have higher proportions of donor-reactive CD137-expressing T cells prior to kidney transplantation

CD137 is expressed at the cell surface of T cells upon the interaction of the T-cell receptor with antigen presented by antigen-presenting cells in the context of HLA class I (CD8+ T cells) or HLA class II (CD4+ T cells) and allows for identification of antigen-reactive T cells [6, 14–16]. Prior to transplantation, the median (IQR) % of CD4+ and CD8+ T cells expressing CD137 amounted to 0.11 (0.07–0.15)% and 1.29 (0.48–2.59)% following donor–antigen stimulation, respectively. The median background signal (without stimulation) amounted to 0.04 (0.02–0.07)% and 0.27 (0.05–0.49)% for CD137-expressing CD4+ and CD8+ T cells, respectively. Percentages CD137-expressing (“CD137total”) T cells before transplantation for rejectors and non-rejectors were 0.13 (0.08–0.20)% versus 0.10 (0.07–0.14)% and 1.72 (0.73–3.30)% versus 0.91 (0.40–1.72)% for CD4+ (*P* = 0.05; Fig. 1B) and CD8+ (*P* < 0.01; Fig. 1D) T cells, respectively. Within the CD137 total-expressing CD4+, as well as CD8+, T cells, a proportion of cells with high expression of CD137 (“CD137++”) was observed (Fig. 1C and E), that was significantly higher (by as much as 50–80%; *P* < 0.01) in rejectors versus non-rejectors. Percentages CD137++ amounted to 0.03 (0.02–0.04)% versus 0.02 (0.01–0.03)% and 0.18 (0.08–0.40)% versus 0.10 (0.05–0.21)% for CD4+ and CD8+ T cells, respectively. In conclusion, before kidney transplantation, a higher frequency of donor-reactive CD137-expressing CD4+ and CD8+ T cells was observed in the group of recipients who experienced aTCMR after transplantation compared to patients who remained rejection-free. This difference was most pronounced for the CD137++ subset.

### Rejectors have higher proportions of cytokine-producing CD137++ CD4+ and CD8+ T cells prior to transplantation

Cytokine-producing T cells were enriched within the CD137++ compared to CD137 total-expressing donor-reactive T-cell fraction, i.e. median frequencies of total cytokine-producing cells amounted to 50% versus 30.3% and 21.8% versus 7.3% for CD137++ versus CD137total CD4+ (Supplementary Fig. S1A) and CD8+ (Supplementary Fig. S1B) T cells, respectively. Both single and polyfunctional cytokine-producing T cells were increased within this subset of CD137++ compared to CD137 total-expressing T cells. Frequencies amounted to 27.3% versus 19.1% and 20.6% versus 10.0% for CD137++ versus CD137 total-expressing single and polyfunctional cytokine-producing CD4+ T cells, respectively (Supplementary Fig. 1A). In addition for CD8+ T cells, frequencies amounted to 17.2% versus 6.3% and 3.8% versus 0.8% for CD137 high versus CD137 total-expressing single and polyfunctional cytokine-producing cells, respectively (Supplementary Fig. S1B).

Donor-reactive CD137++ CD4+ T cells were able to produce IL-2, IFN-γ, and TNF-α simultaneously, and this proportion amounted to approximately 15% of the polyfunctional T cells. The minority of donor-reactive CD137++ CD8+ T cells produced IL-2, therefore hardly any triple cytokine-producing cells were noted. Most of the polyfunctional donor-reactive CD137++ T cells produced two pro-inflammatory cytokines simultaneously (>80%). For CD4+ T cells, the predominant fraction consisted of IL-2 and TNF-α (i.e. amounting to approximately 64–69% of polyfunctional T cells) whereas, for CD8+ T cells, most T cells produced IFN-γ and TNF-α (i.e. amounting to approximately 80–90% of polyfunctional T cells).

**Figure 2.**
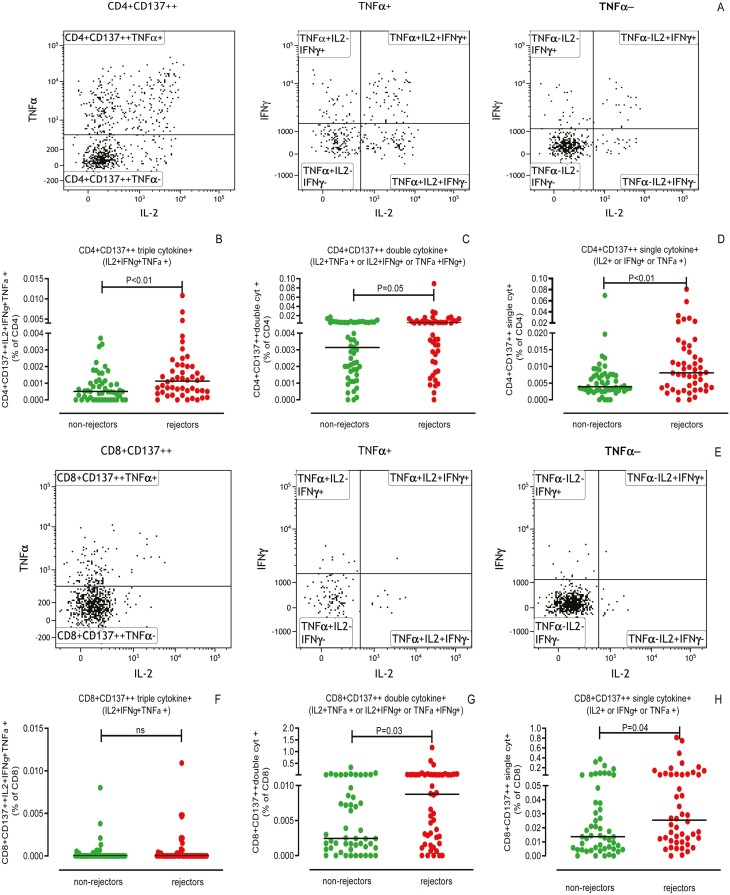
Cytokine-producing donor-reactive CD137++ CD4+ and CD8+ T cells. A typical flow cytometric example of the identification of polyfunctional (triple, double) as well as single cytokine-producing donor-reactive CD137++ CD4+ and CD8+ T cells is given in **A** and **E**, respectively. Briefly, CD137++ T cells are plotted in a dotplot displaying TNF-α on Y-axis and IL-2 on X-axis to identify TNF-α+ and TNF-α− cells. These are subsequently dissected using a dotplot depicting IFN-γ on Y-axis and IL-2 on X-axis in order to determine the different proportions of cytokine producing cells. Proportions of different types of polyfunctional (triple and double) and single cytokine producing cells prior to kidney transplantation are depicted as a % of CD4+ (**B–D**) and CD8+ (**F–H**) T cells, respectively. Recipients experiencing a TCMR within the first year after kidney transplantation are depicted (rejectors) and those without (non-rejectors). The horizontal line depicts the median value. *P* < 0.05 is statistically significant

Donor-reactive CD4+ T cells of rejectors contained higher proportions of CD137++ polyfunctional cytokine-producing cells and single cytokine-producing cells than non-rejectors prior to transplantation (*P* < 0.01; Fig. 2C). Evaluation of the combination of cytokines revealed that those producing IFN-γ, IL-2, and TNF-α (*P* < 0.01; Fig. 2A) simultaneously, as well as those producing IL-2 or TNF-α combined with IFN-γ (*P* < 0.01) to be the most significantly different. Proportions of polyfunctional (*P* = 0.03) and single cytokine-producing CD137++ cells were also significantly (*P* = 0.04; Fig. 2H) higher within the CD8+ T-cell fraction prior to transplantation in rejectors compared to non-rejectors. Those producing IL-2 and TNF-α (*P* = 0.02) were the most significantly different.

Next, we evaluated whether proportions of the degranulation marker CD107a-expressing donor-reactive CD137++ T cells in combination with these pro-inflammatory cytokines allowed for a better discrimination between rejectors and non-rejectors. A significantly higher proportion of donor-reactive CD137++ cells, lacking TNF-α and IFN-γ, but positive for IL-2 and CD107a, was observed for both CD4+ (*P* = 0.02) and CD8+ (*P* < 0.05) T cells. In conclusion, inclusion of CD107a-expressing cells did not result in a better discrimination of recipients with a biopsy-proven aTCMR (data not shown).

In addition, proportions of polyfunctional donor-reactive CD137++ T cells were neither associated with the % PRA nor with number of mismatches for HLA I or II (data not shown).

### Multivariate analysis of clinical and demographic characteristics of study population

Significant different clinical and demographic characteristics (Table 1) between recipients experiencing an aTCMR within the first year after transplantation and those that remain free of rejection, were tested in a multivariate analysis. Neither recipient age (Pillai’s Trace multivariate test, *P* = 0.47) nor donor age (Pillai’s Trace multivariate test, *P* = 0.23) did contribute to the variation observed but type of transplantation did (Pillai’s Trace multivariate test, *P* = 0.03). Pre-transplant proportions of polyfunctional (including triple as well as double) as well as single cytokine producing donor-reactive CD4+ and CD8+ T cells were higher for rejectors versus non-rejectors when zooming in to the living but not deceased donor kidney transplant recipients (Table 2).

**Table 2.** Proportions of donor-reactive cytokine producing CD137++ CD4 and CD8+ T cells for recipients receiving living and deceased donor kidneys

	Living donor			Deceased donor		
CD137++ population of cyt pc ^*^	Rejector (*N* = 30)	Non-rejector (*N* = 40)	*P* value	Rejector (*N* = 18)	Non-rejector (*N* = 11)	*P* value
CD4+ polyfunctional (%)	0.006	0.004	0.015	0.004	0.004	0.669
CD4+ triple+ (%)	0.001	0.000	0.003	0.001	0.001	0.573
CD4+ double+ (%)	0.005	0.003	0.028	0.004	0.003	0.544
CD4+ single+ (%)	0.008	0.004	0.001	0.010	0.005	0.719
CD8+ polyfunctional (%)	0.007	0.002	0.024	0.011	0.012	0.753
CD8+ triple+ (%)^**^						
CD8+ double+ (%)	0.006	0.002	0.025	0.010	0.012	0.753
CD8+ single+ (%)	0.022	0.010	0.017	0.026	0.021	0.787

^*^cyt pc, cytokine producing cells; % are of CD4+ and CD8+ T cells, respectively.

^**^CD8+ triple cytokine-producing cells were hardly detected and not included in this table.

Median values are depicted and values for R and NR are compared using the Mann–Whitney *U* test, *P* < 0.05 is considered statistically significant.

### Phenotypic characteristics of (polyfunctional) donor-reactive CD137++ T cells

**Figure 3.**
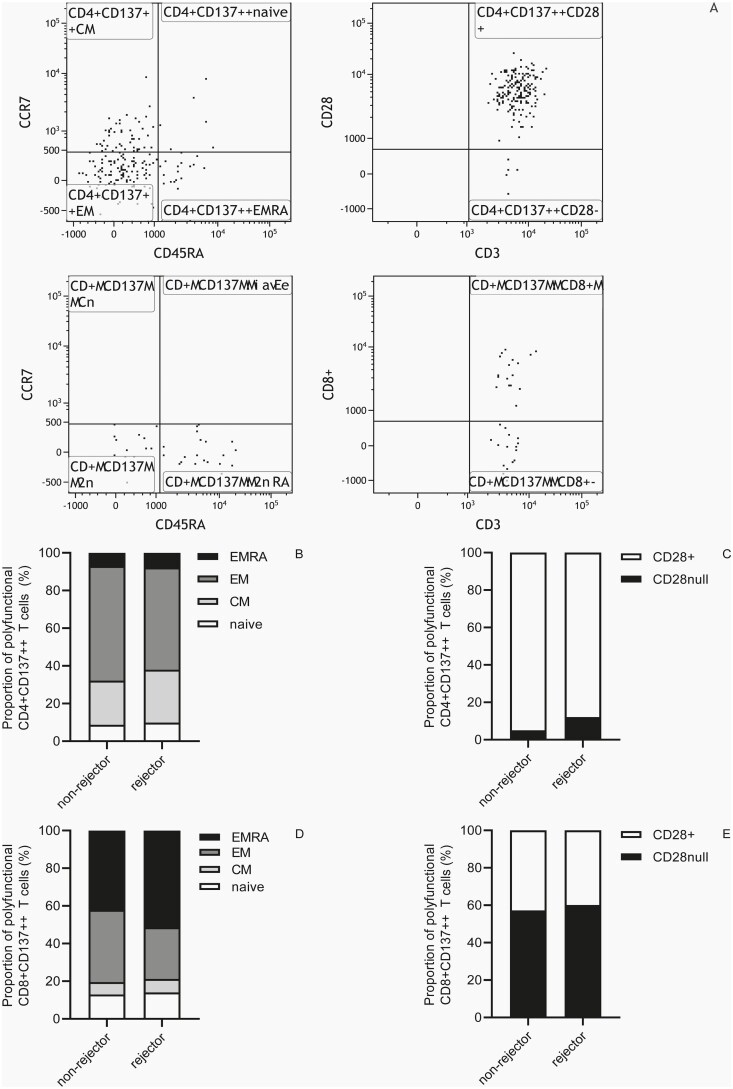
Phenotype of polyfunctional donor-reactive CD137++ T cells. A typical flow cytometric example of the phenotypic characteristics of polyfunctional donor-reactive T cells prior to kidney transplantation is given in **A**. Briefly, CD137++ T cells producing two or more pro-inflammatory cytokines were dissected into T-cell subsets using antibodies directed to CD45RA, CCR7, and CD28. The top panel depicts polyfunctional donor-reactive CD137++ CD4+ and the bottom panel CD8+ T cells. Naïve T cells were identified as CD45RA+CCR7+, central memory (CM) as CD45RA−CCR7+, effector memory (EM) as CD45RA−CCR7− and terminally differentiated effector memory (EMRA) T cells as CD45RA+CCR7−. In addition, less differentiated polyfunctional T cells were identified as co-expressing CD28 whereas the more differentiated ones as lacking CD28 (CD28−, CD28null). Characterization of phenotype of polyfunctional donor-reactive CD137++ CD4+ T cells for non-rejectors and rejectors is given as stacked bars in **B** and **C** whereas that of CD8+ T cells is given in **D** and **E**. Each sub-bar represents the mean

Pre-transplant phenotypic characteristics of donor-reactive CD137++ CD4+ (Supplementary Fig. 2A and B) as well as CD137++CD8+ (Supplementary Fig. 2C and D) T cells were not different between recipients experiencing an aTCMR within the first year after transplantation and those that remained free of a rejection. In addition, the phenotype of polyfunctional donor-reactive CD137++ T cells before transplantation was also not different between kidney transplant recipients developing an aTCMR and those that remained free of rejection. Prior to transplantation, polyfunctional donor-reactive CD137++ CD4+ T cells were predominantly of the CM and EM (Fig. 3B) phenotype, co-expressing CD28 (Fig. 3C). Mean (95%CI) frequencies CM and EM polyfunctional donor-reactive CD137++ CD4+ T cells amounted to 28.1 (21.9–34.2)% and 54.3 (47.3–61.4)% versus 23.4 (17.3–29.6)% and 60.8 (52.5–69.2)% for rejectors and non-rejectors, respectively (Fig. 3B). In addition, frequencies CD28-co-expressing polyfunctional donor-reactive CD137++ CD4+ T cells amounted to 87.8 (80.3–95.4)% versus 95.0 (90.1–99.8)% for rejectors and non-rejectors, respectively (Fig. 3C). Polyfunctional donor-reactive CD137++CD8+ T cells were of EM and more differentiated EMRA (Fig. 3D) phenotype and approximately 40% of them co-expressed CD28 (Fig. 3E). Mean (95%CI) frequencies EM and EMRA polyfunctional donor-reactive CD137++ CD8+ T cells amounted to 27.4 (17.8–37.0)% and 51.4 (41.5–61.2)% versus 38.3 (26.3–50.3)% and 42.1 (29.0–55.3)% for rejectors and non-rejectors, respectively (Fig. 3B). In addition, frequencies of CD28-co-expressing polyfunctional donor-reactive CD137++ CD8+ T cells amounted to 39.9 (27.9–51.9)% versus 42.8 (27.2–58.4)% for rejectors and non-rejectors, respectively (Fig. 3E).

### Proportions of pre-transplant polyfunctional donor-reactive CD137++ T cells are not related to the type of aTCMR

**Figure 4.**
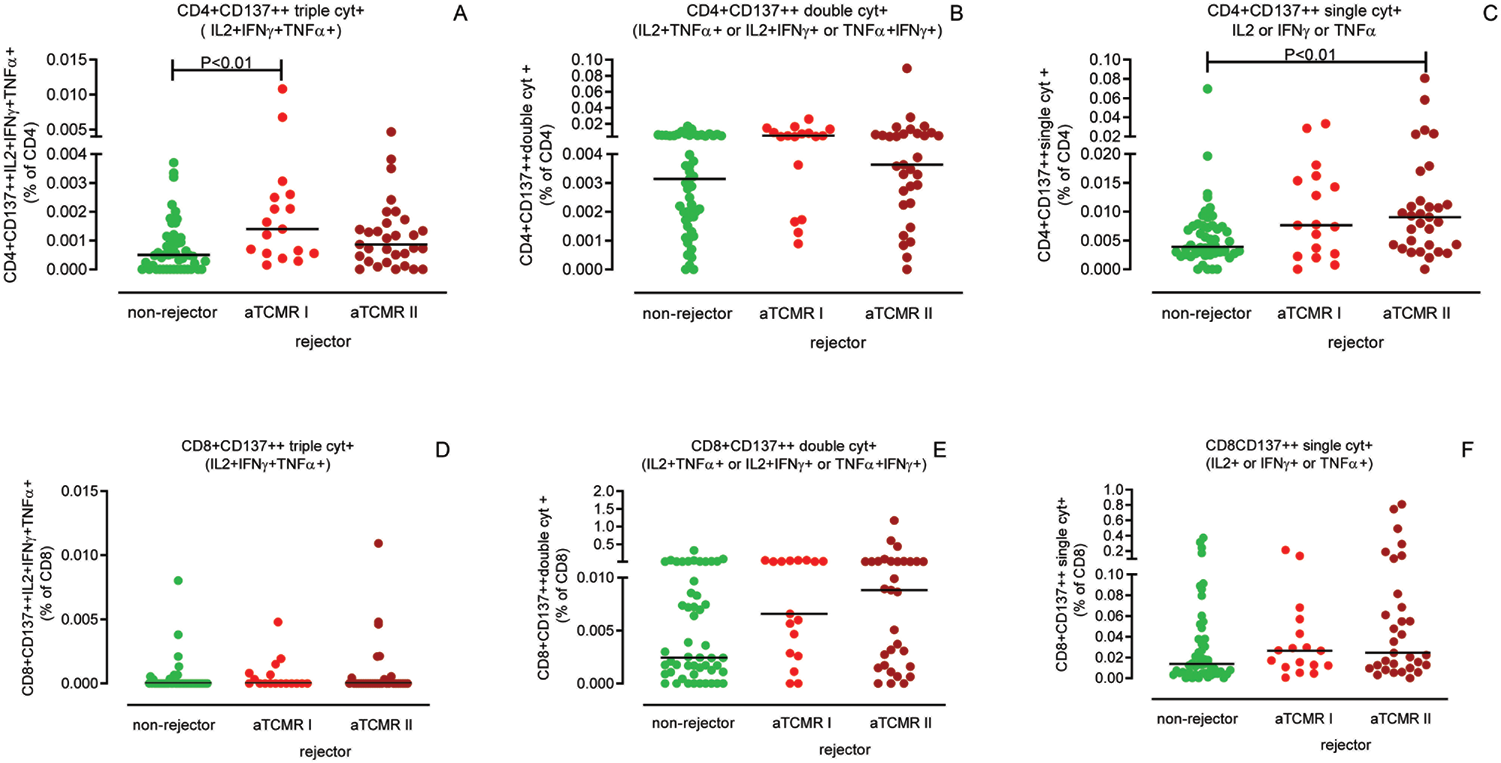
Cytokine-producing donor-reactive CD137++ CD4+ and CD8+ T cells are not discriminative for type of aTCMR. Biopsies of kidney transplant recipients developing an aTCMR within the first year after transplantation, were subdivided according to Banff criteria in aTCMR I and the more severe aTCMR II. Proportions of different types of polyfunctional (triple and double) and single cytokine-producing donor-reactive CD137++ T cells prior to transplantation are depicted as a % of CD4+ (**A–C**) and CD8+ (**D–F**) T cells, respectively. Seventeen recipients developed a biopsy-proven aTCMR I, 32 developed an aTCMR II and 51 remained free of a rejection within the first year after kidney transplantation. The horizontal line depicts the median value. *P* < 0.05 is statistically significant

Polyfunctional (Fig. 4A, B, D and E) as well as single cytokine producing (Fig. 4C and F) donor-reactive CD137++ CD4+ and CD8+ T cells could not discriminate between rejectors experiencing an aTCMR I and those experiencing the more severe, vascular, type of aTCMR (aTCMR II). Proportions of triple (Fig. 4A), IL-2 and IFN-γ and IFN-γ and TNF-α producing donor-reactive CD137++ CD4+ T cells were higher prior to transplantation in recipients diagnosed with an aTCMR I than non-rejectors. In addition, proportions of single cytokine producing donor-reactive CD137++ CD4+ T cells were higher for recipients experiencing vascular type of TCMR when compared to those who remained free of rejection (Fig. 4C). Within the donor-reactive CD137++ CD8+ T cell fraction, proportions of IL-2 and TNF-α-producing cells were higher in individuals diagnosed with an aTCMR I compared to those without a rejection within the first year after transplantation but again not different from those diagnosed with an aTCMR II.

### The frequency of circulating polyfunctional donor-reactive CD137++ CD4+, but not CD137++ CD8+, T cells decreases with time after transplantation but is not related to rejection

**Figure 5.**
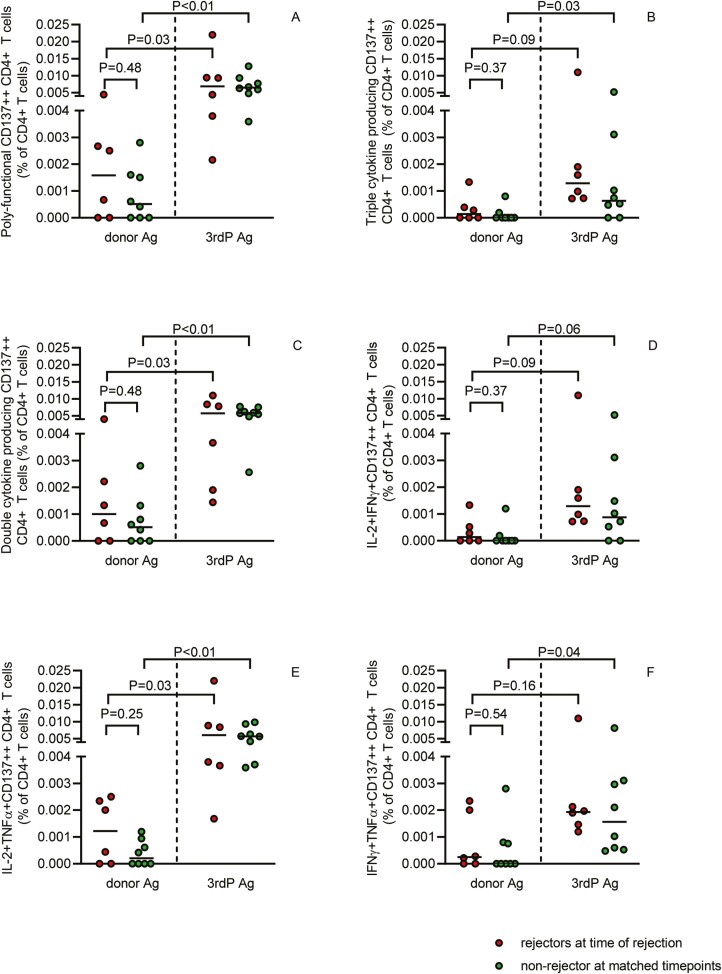
Polyfunctional donor-reactive CD137++ CD4+ T cells at time of a for-cause biopsy. At time of a biopsy-proven aTCMR (rejectors; red circles; *N* = 6) and at matched time points after transplantation (non-rejectors; green circles; *N* = 4, of which two at multiple time-points), polyfunctional donor-reactive CD137++ CD4+ T cells (left side of graph) were measured. In addition, polyfunctional 3rd P-reactive CD137++ T cells (right side of the graph) were measured. Proportions of different types of polyfunctional cytokine-producing donor-reactive CD137++ T cells are depicted as a % of CD4+ (**A–F**). *P* < 0.05 is statistically significant

Next, polyfunctional donor-reactive CD137++ T cells at time of a biopsy-proven aTCMR in 7 kidney transplant recipients (rejectors) were compared to non-rejectors at matched time points after transplantation. Both rejectors and non-rejectors, had lower proportions of polyfunctional (Fig. 5A), double (Fig. 5C), and in particular IL-2+TNFα+ (Fig. 5E), compared to 3rd P-reactive CD137++ CD4+ T cells. The decrease over time for proportions of polyfunctional, double, and IL-2+TNF-α+ cytokine producing cells was not different for rejectors and non-rejectors and amounted to 73%, 81%, 82%, and 77%, 66%, and 91%, respectively. Donor-reactive polyfunctional CD137++ CD8+ T cells did not significantly decrease in rejectors and non-rejectors and remained at a similar % as 3rd P-reactive CD137++ CD8+ T cells (results not shown).

## Discussion

The main finding of this study is that higher numbers of CD137++ polyfunctional donor-reactive T cells in recipients prior to kidney transplantation are associated with the development of aTCMR within the first year after transplantation.

Three to five years after transplantation, the risk for an aTCMR declines. We have previously described that loss of polyfunctional CD137-expressing donor-reactive CD4+ T cells was associated with donor-specific hypo-responsiveness occurring 3–5 years after transplantation [7, 9]. Our data fit the data of the previous studies, in that polyfunctional donor-reactive CD4+ T cells constitute a risk factor for acute rejection.

CD137 is a co-stimulatory molecule upregulated on T cells upon interaction of the T cell receptor (TCR) with HLA on antigen-presenting cells and can be used to identify alloreactive T cells. The current data show that a subset of alloreactive T cells expressing high levels of CD137 can be identified, harboring functional (cytokine-producing) T cells, which associates with the risk for rejection. T cells highly expressing CD137 might reflect a stronger interaction between T cells and antigen-presenting cells, i.e. a more activated T cell. High avidity TCR donor-reactive T cells have indeed been associated with acute rejection [17]. Not only polyfunctional but also single cytokine-producing donor-reactive CD137++ T cells were higher for rejectors when compared to non-rejectors. This finding indicates that high expression of the costimulatory molecule CD137 together with cytokine production may be more relevant than polyfunctionality *per se*.

Prior to transplantation, the measured donor-reactivity is mostly due to cross-reacting memory T cells specific for viruses [18]. Characteristics of memory T cells include enhanced responses compared to naïve T cells [19]. Prior to transplantation, polyfunctional donor-reactive CD137++ T cells were mostly of the EM- and, in particular for CD8+ T cells, EMRA-phenotype. These cells are able to migrate to peripheral tissues [20], i.e. to the renal allograft to cause harm. CD4+ T cells mostly co-expressed CD28, whereas only half of the CD8+ T cells did. This was also described previously by our group [21] and others [22]. After transplantation, naïve T cells may also respond to donor-antigen and develop into memory T cells, thereby generating another pool of T cells able to attack the donor organ. However, de novo responses by naïve T cells to donor-antigens are more susceptible to immunosuppression compared to memory T cells [23, 24], implying that the latter is more dangerous to the graft.

A recent paper by Xie et al. [25]. illustrated increased polyfunctionality of alloreactive T cells to be linked to allograft pathologies, including rejection. They examined cytokine secretion within proliferating T cells measured using single-cell proteome analysis. Flow-cytometry-based assays have already been used before for estimating the alloreactive potential but the functional profile was limited to evaluating only one cytokine, i.e. IFN-γ [22, 26, 27]. Ortiz et al. did measure polyfunctional alloreactive T cells by analyzing frequencies of IL-2, IFN-γ, and TNF-α producing cells using flow cytometry [28]. However, they used a 6-day stimulation protocol, of which the last 4 h were in presence of a cytokine secretion inhibitor. Due to the 6-day-stimulation period, cells might have differentiated from naïve and/or central memory to an effector memory phenotype and the cytokine profile may have changed as well. We have examined the cytokine profile and phenotype of donor-reactive T cells following a short-term stimulation, with minimal to no effects on T-cell phenotype allowing a more accurate evaluation of donor-reactive T cells. The polyfunctional profile differed for CD4+ and CD8+ donor-reactive T cells. Whereas donor-reactive CD137++ CD4+ T cells were able to produce all three pro-inflammatory cytokines examined, most of the polyfunctional donor-reactive CD137++ CD4+ T cells were producing two, i.e. IL-2 and TNF-α, simultaneously. In contrast, donor-reactive CD137++ CD8+ T cells were hardly able to produce IL-2, IFN-γ, as well as TNF-α simultaneously. They pre-dominantly produced IFN-γ and TNF-α as described before [29]. Although IL-2 can be produced by CD8+ T cells, IL-2 producing CD8+ T cells are mainly confined to the CM subset and donor-reactive CD8+ T cells mainly belonged to the EM and EMRA subsets. This might explain the fact that hardly any triple cytokine producing CD8+ T cells were found. In addition, inclusion of other cytokines/effector molecules like for example MIP-1α, MIP-1β or granzymes and perforins specific for donor-reactive CD137++ CD8+ T cells might have resulted in differences in the proportion of CD8+ T cells capable of producing three factors. In this respect, inclusion of the degranulation marker CD107a in our study did not allow for a better characterization of polyfunctional donor-reactive CD8+ T cells. TNF-α was shown to be a prominent cytokine produced by donor-reactive T cells in our study. TNF-α plays a role in allograft rejection [30–32] and elevated levels were found in recipients experiencing acute rejection of the renal allograft [33]. In addition, TNF was also suggested as target for therapy in organ transplantation [34].

Implementation of this short-term stimulation multi-parameter flow cytometry-based CD137-assay to predict the risk for an aTCMR prior to transplantation is difficult as there is a large overlap between individuals who develop an aTCMR and those that do not. This finding is generally true for most publications on this topic, in particular the IFN-γ ELISPOT assays [3–5]. An earlier attempt in identification of low-immunological risk transplant recipients by absence of pre-transplant donor-specific T cells using the IFNγ ELISPOT assay to minimize immunosuppression was unsuccessful [35].

At the time of biopsy-proven aTCMR, polyfunctional donor-reactive CD4+, but not CD8+ T cells were present in lower numbers in the circulation compared to before transplantation but this was also observed for non-rejectors who were at a similar time intervals after transplantation. This decrease in allo-reactive CD4+ T cells early after transplantation, followed by donor-reactive CD8+ T cells at later time points (>5 years) after transplantation has been previously observed [9]. One might expect a change in the frequency of circulating alloreactive T cells at time of rejection, either showing the expansion of these cells or a decrease in numbers indicating migration into the graft. A recent publication showed that the frequency of circulating alloreactive T-cell clones before transplantation and at time of rejection of the transplanted kidney was similar, although the frequency was increased within the allograft [36]. Taken together, these findings indicate that the kinetics of circulating donor-reactive T cells after transplantation are unlikely to predict acute rejection.

Limitations of the present study include the retrospective design (case–control), prohibiting assessment of the sensitivity and specificity of this multi-parameter flow cytometry-based CD137-assay. In addition, in our cohort, we only matched for a period of transplantation when selecting controls (recipients remaining free of rejection). Both recipient and donor age differed significantly between rejectors and non-rejectors as did the type of transplantation (living or deceased donor kidney). However, a multivariate analysis did not show recipient age as well as a donor age to be a major confounder. The type of transplantation/donor, i.e. living versus deceased donor, did however impact the variation observed. Most of our recipients received a kidney from a living donor and in this cohort higher proportions of (cytokine producing) donor-reactive CD137++ T cells were associated with an aTCMR within the first year after transplantation. Significant differences between rejectors and non-rejectors were lost when considering deceased donor kidney recipients. Currently, we cannot explain this and more research is warranted to validate these findings in a larger cohort of deceased donor transplant recipients. Moreover, we selected three pro-inflammatory cytokines and did not measure other cytokines known to play a role in allograft rejection such as IL-17 [37, 38] or IL-21 [39]. However, Th17 cells, producing IL-17, have been implicated in allograft rejection in heart transplant recipients although their role in TCMR in kidney transplant recipients is less clear. In addition, as IL-21 was described to be predominantly produced by follicular T helper (Tfh) cells, a cell type associated with antibody-mediated rejection, we did not include this cell type in our study.

In conclusion, frequencies of pre-transplant donor-reactive T cells with high CD137-expression and producing one or more cytokines are higher in kidney transplant recipients developing an aTCMR within the first year after transplantation compared to recipients who remain free of rejection. These findings underline the importance of these cells in the direct alloreactive T-cell response against the transplanted kidney.

## Supplementary Material

uxad041_suppl_Supplementary_Figure_S1

uxad041_suppl_Supplementary_Figure_S2

uxad041_suppl_Supplementary_Table_S1

## Data Availability

The data underlying this article will be shared on reasonable request to the corresponding author.

## Abbreviations

AIM: activation-induced marker
APC (Cy7/H7): allophycocyanin
aTCMR: acute T-cell mediated rejection
aTCMR I: tubulo-interstitial aTCMR
aTCMR II: vascular aTCMR
BV: brilliant violet
CD: cluster of differentiation
CM: central memory
ELISPOT: anzyme-linked immunospot
EM: effector memory
EMRA: terminally differentiated CD45RA+ effector memory
FITC: fluorescein isothiocyanate
FVS: fixeable viability stain
HLA: human leukocyte antigen
IFN-γ: interferon-gamma
IL-: interleukin
IQR: Interquartile range
MEC: medical ethical committee
MIP-1 (α or β): macrophage inflammatory protein
PBMCs: peripheral blood mononuclear cells
PE(Cy7): phycoerythrin
PERCP-Cy5.5: peridinin-chlorophyll-protein
PRA: panel reactive antibodies
TNF-α: tumor-necrosis factor alpha
