# Supplementary figures and images for: Polyfunctional donor-reactive T cells are associated with acute T-cell-mediated rejection of the kidney transplant
Source: Clin Exp Immunol. 2023 Apr 18;213(3):371–83. doi: 10.1093/cei/uxad041 (PMC10571010; doi:10.1093/cei/uxad041)

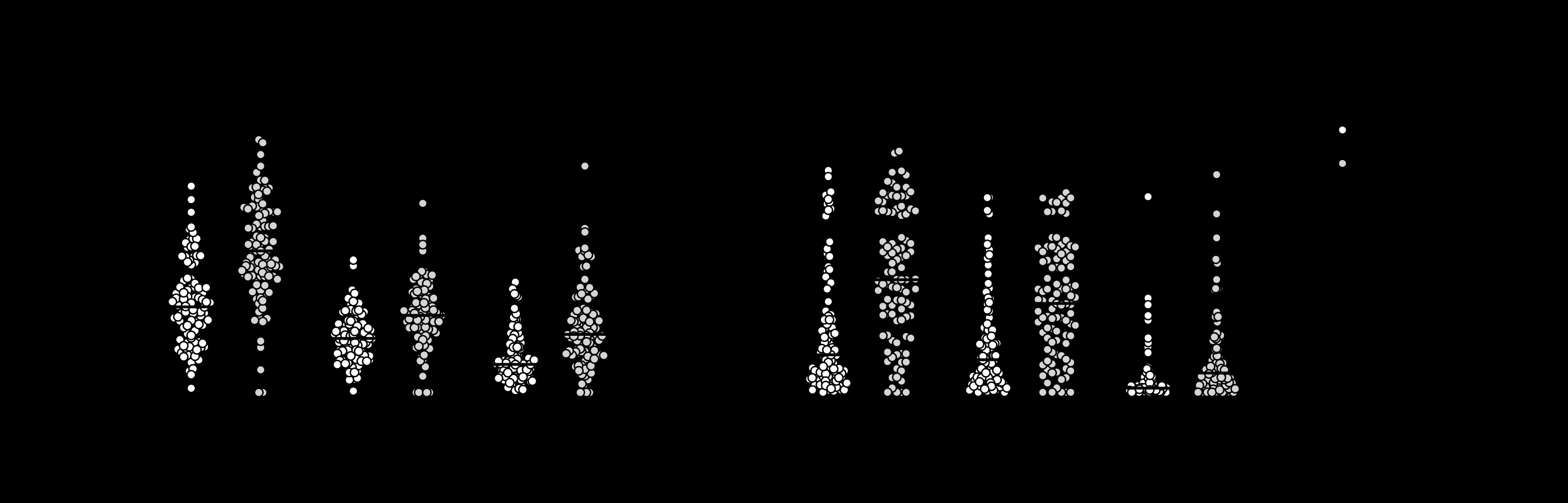

Supplement: uxad041_suppl_Supplementary_Figure_S1 [file uxad041_suppl_supplementary_figure_s1.jpeg]

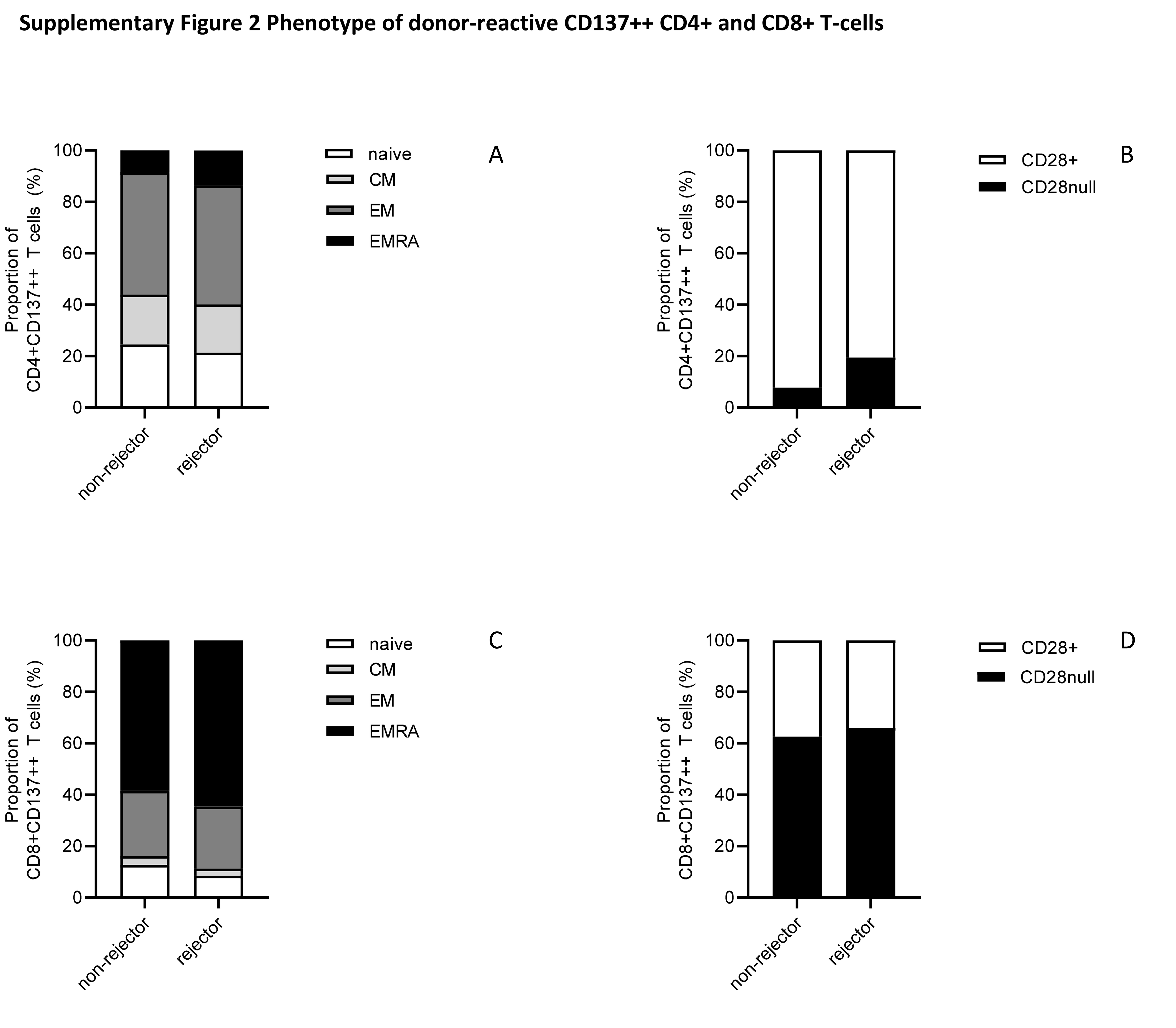

Supplement: uxad041_suppl_Supplementary_Figure_S2 [file uxad041_suppl_supplementary_figure_s2.jpeg]
