# Supplementary material for: Polyfunctional donor-reactive T cells are associated with acute T-cell-mediated rejection of the kidney transplant
Source: Clin Exp Immunol. 2023 Apr 18;213(3):371–83. doi: 10.1093/cei/uxad041 (PMC10571010; doi:10.1093/cei/uxad041)
Supplement: uxad041_suppl_Supplementary_Table_S1 [file uxad041_suppl_supplementary_table_s1.docx]

**Supplementary Table 1 Monoclonal antibodies**

| **Marker** | **Fluorochrome** | **Company** | **Clone** | **Cell surface (s) /intracellular (i)** |
| --- | --- | --- | --- | --- |
| CD137 | APC | BD | 4B4-1 | I |
| CD3 | AlexaFluor (AF)700 | Biolegend | HIT3a | S |
| CD4 | PerCPCy5.5 | BD | RPA-T4 | S |
| CD8 | BV510 | Biolegend | RPA-T8 | S |
| CD14 | APC-H7 | BD | MφP9 | S |
| CD19 | APC-H7 | BD | SJ25C1 | S |
| CD56 | APC-Cy7 | BioLegend | HCD56 | S |
| FVS780 |  | BD | none | S |
| CD45RA | BV785 | BioLegend | HI100 | S |
| CCR7 | PE | BioLegend | G043H7 | S |
| CD28 | PECy7 | BioLegend | CD28.2 | S |
| CD107a | FITC | Biolegend | H4A3 | present during stimulation |
| IL-2 | BV421 | BD | MQ-17H12 | I |
| IFN-γ | BV711 | BD | 4SB3 | I |
| TNF-α | BV605 | BD | Mab11 | I |

Becton Dickinson(BD), Erembodegem, Belgium

Biolegend, Amsterdam, the Netherlands
